# Linking Inter-Individual Variability in Functional Brain Connectivity to Cognitive Ability in Elderly Individuals

**DOI:** 10.3389/fnagi.2017.00385

**Published:** 2017-11-21

**Authors:** Rui Li, Shufei Yin, Xinyi Zhu, Weicong Ren, Jing Yu, Pengyun Wang, Zhiwei Zheng, Ya-Nan Niu, Xin Huang, Juan Li

**Affiliations:** ^1^CAS Key Laboratory of Mental Health, Institute of Psychology, Beijing, China; ^2^Department of Psychology, University of Chinese Academy of Sciences, Beijing, China; ^3^Department of Psychology, Faculty of Education, Hubei University, Wuhan, China; ^4^Department of Education, Hebei Normal University, Shijiazhuang, China; ^5^Faculty of Psychology, Southwest University, Chongqing, China; ^6^Magnetic Resonance Imaging Research Center, Institute of Psychology, Chinese Academy of Sciences, Beijing, China; ^7^State Key Laboratory of Brain and Cognitive Science, Institute of Biophysics, Chinese Academy of Sciences, Beijing, China

**Keywords:** individual variability, functional connectivity, cognitive aging, fMRI, brain networks

## Abstract

Increasing evidence suggests that functional brain connectivity is an important determinant of cognitive aging. However, the fundamental concept of inter-individual variations in functional connectivity in older individuals is not yet completely understood. It is essential to evaluate the extent to which inter-individual variability in connectivity impacts cognitive performance at an older age. In the current study, we aimed to characterize individual variability of functional connectivity in the elderly and to examine its significance to individual cognition. We mapped inter-individual variability of functional connectivity by analyzing whole-brain functional connectivity magnetic resonance imaging data obtained from a large sample of cognitively normal older adults. Our results demonstrated a gradual increase in variability in primary regions of the visual, sensorimotor, and auditory networks to specific subcortical structures, particularly the hippocampal formation, and the prefrontal and parietal cortices, which largely constitute the default mode and fronto-parietal networks, to the cerebellum. Further, the inter-individual variability of the functional connectivity correlated significantly with the degree of cognitive relevance. Regions with greater connectivity variability demonstrated more connections that correlated with cognitive performance. These results also underscored the crucial function of the long-range and inter-network connections in individual cognition. Thus, individual connectivity–cognition variability mapping findings may provide important information for future research on cognitive aging and neurocognitive diseases.

## Introduction

There is a marked heterogeneity in cognitive functioning during late adulthood and old age (Hedden and Gabrieli, 2004; Lustig et al., 2009; Nyberg et al., 2012). Some older people may display rapid cognitive decline or develop Alzheimer’s disease (AD), whereas others may continue to exhibit a superior level of cognitive functioning. One of the main contributions to this heterogeneity originates from the variability of the brain (Hedden and Gabrieli, 2004; Reuter-Lorenz and Lustig, 2005; Bishop et al., 2010; Grady, 2012; Tomasi and Volkow, 2012; Karama et al., 2014), particularly in regard to functional connectivity (Burke and Barnes, 2006; Andrews-Hanna et al., 2007; Bishop et al., 2010; Grady, 2012; Tomasi and Volkow, 2012; Ferreira and Busatto, 2013; Fornito et al., 2015).

Previous studies demonstrated that preserved functional integration between distributed brain regions supports proficient cognitive function, while functional disruption of the inter-regional neural communication results in cognitive decline and AD (Andrews-Hanna et al., 2007; Eyler et al., 2011; Grady, 2012; Nyberg et al., 2012; Dennis and Thompson, 2014). Much of this evidence comes from direct comparisons of functional connectivity between groups that are pre-defined by neuropsychological questionnaires or clinical classifications of mental states. For instance, elderly individuals who performed better on a verbal fluency test demonstrated stronger connections between the precuneus and prefrontal regions compared to that in individuals with lower verbal fluency test performance (Yin et al., 2015). Similarly, patients with AD exhibited disrupted functional connectivity in the default mode and several fronto-parietal attention networks, compared to that of healthy elderly individuals (Wang et al., 2007, 2015; Buckner et al., 2009; Myers et al., 2014). These “group differences” provide substantial insights into the brain connectivity correlates of cognitive aging. However, a fundamental issue regarding how functional brain connectivity itself differs among older individuals remains to be elucidated. Although many group-based investigations usually included individual-level results, the “individual difference” in functional connectivity remains largely uninvestigated. For example, Betzel et al. (2014) demonstrated the trajectory of individual functional connectivity of resting state networks with age (Betzel et al., 2014). Similarly, there are studies that have largely demonstrated individual-level correlations between functional connectivity and cognitive performance in normal elderly people (Andrews-Hanna et al., 2007; Sala-Llonch et al., 2014; Yin et al., 2015) and patients (Wang et al., 2015). However, it is still not clear how inter-individual variability in functional connectivity can vary in different brain regions and to what extent the inter-individual variability in connectivity impacts cognitive performance at an older age.

An important reason for the bias toward group differences is that traditional task-based neuroimaging studies are limited in their ability to systematically quantify individual brain function differences, given the diverse nature of the tasks used in different studies. Resting-state functional connectivity magnetic resonance imaging (fcMRI) that measures the intrinsic temporal synchronization of the blood oxygen level-dependent (BOLD) signals has been developed to delineate the neural functional architecture in human participants who are not engaged in any specific task. Similar to genomic and phenomic approaches, fcMRI is recognized as a remarkably powerful tool to understand individual variation in brain functioning (Mohr and Nagel, 2010; Buckner, 2013; Mueller et al., 2013; Zatorre, 2013). Mueller et al. (2013) recently measured individual differences of the resting-state connectivity of the cortical regions in 25 healthy adults. The authors reported higher variability in the association cortex and lower variability in the unimodal cortices. Similarly, Gao et al. (2014) examined the inter-individual variability of functional connectivity during infancy (Gao et al., 2014). However, to our knowledge, there have been no studies to date regarding the distribution of the inter-individual differences in functional connectivity in the brains of elderly individuals.

In the current study, we aimed to investigate two major issues as follows: (1) we sought to delineate the inter-individual variability map of functional brain connectivity during old age. The fcMRI data from 108 healthy older adults were collected during resting-state conditions. The brain was divided into 116 regions of interest (ROIs), including cortical, subcortical, and cerebellar regions, using the automated anatomical labeling (AAL) procedure (Tzourio-Mazoyer et al., 2002). The variation of the individual-to-individual functional connectivity in each ROI of these older adults were then estimated and used to generate the brain variability map. Further, to facilitate the inspection of the brain distribution for the inter-individual variability, the variability was compared in 6 distinct brain systems, including the default mode, fronto-parietal, visual, sensorimotor and auditory, subcortical, and cerebellar networks (Ferrarini et al., 2009; He et al., 2009); and 2) we then linked the inter-individual variability of the connectivity to cognitive function in the elderly. A battery of standardized neuropsychological tests was employed to assess the cognitive function of the older participants. The connectivity–cognition association was first examined by calculating the correlations between each region’s connectivity and the cognitive test performance. This allowed us to determine whether the regions that had correlations between connectivity and cognitive ability were concentrated in the areas with large inter-individual variability for functional connectivity. Then, we defined a cognitive relevance index that was calculated as the number of cognition-correlated connections to quantify the role of each region’s functional connectivity in cognitive functioning. To examine the cognitive significance of the distribution of inter-individual variability of functional connectivity, a correlation between the value of inter-individual variability and the degree of cognitive relevance was computed across all ROIs. This allowed us to further determine whether regions with larger inter-individual variability in the brain connectivity would play a more important role in cognitive performance of the elderly. Recent studies have suggested that the long-range and inter-network regional connections function critically in cognitive processing and cognitive aging (Tomasi and Volkow, 2012; Park and Friston, 2013; Fjell et al., 2015). Therefore, to better describe the relationship between variability in connectivity to cognitive significance, we also investigated whether this relationship was more specific to the long-range and inter-network connections.

## Materials and Methods

### Participants

A total of 108 cognitively normal, older volunteers (70.3 ± 5.7 years; range: 60–80 years of age; 50 men and 58 women) were recruited from communities near the Institute of Psychology-Chinese Academy of Sciences. All participants met the following inclusion criteria: age ≥60 years; a score ≥ 21 on the Beijing Version of the Montreal Cognitive Assessment (Yu et al., 2012); a score ≤ 16 on the Activities of Daily Living (Lawton and Brody, 1969); right-handed; and free of stroke, heart disease, diabetes mellitus, neurological and psychiatric disorders, and traumatic brain injury. The images were collected under resting-state conditions using a 3.0-T Siemens Trio scanner (Erlangen, Germany), located at the Beijing MRI Center for Brain Research. Functional imaging consisted of 33 T2^∗^-weighted echo-planar image (EPI) slices (time repetition (TR) = 2000 ms, time echo (TE) = 30 ms, flip angle = 90°, field of view (FOV) = 200 mm × 200 mm, thickness = 3.0 mm, gap = 0.6 mm, acquisition matrix = 64 × 64, and in-plane resolution = 3.125 × 3.125). We collected 200 functional volumes for each participant. T1-weighted anatomical images were collected using a magnetization-prepared rapid gradient echo (MPRAGE) sequence (176 slices, acquisition matrix = 256 × 256, voxel size = 1 mm × 1 mm × 1 mm, TR = 1900 ms, TE = 2.2 ms, and flip angle = 9°) for co-registration with the functional images. Of the total number of participants, 85 participants completed a battery of neuropsychological assessments, which included the Digit Forward Span (DFS) and Digit Backward Span (DBS) (Gong, 1992), the Paired Associative Learning Test (PALT) (Xu and Wu, 1986), the Trail Making Test (TMT) Parts A and B (Reitan, 1986), and the Verbal Fluency Test (VFT) (Rosenberg et al., 1984).

Five participants were excluded due to poor image quality or gross structural abnormalities. Six participants were excluded because of excessive head movements (more than 2.0 mm maximum translation or 2.0° rotation) during the scan. Nine participants were excluded because of bad registration quality during the visual inspection for the normalization. Thus, the final statistical analysis included fMRI data from 88 older adults (70.2 ± 5.6 years; range: 60–80 years of age; 40 men and 48 women). Of these, 76 individuals (70.7 ± 5.5 years; range: 60–80 years of age; 35 men and 41 women) completed the neuropsychological assessments and provided behavioral data.

The institutional review board of the Institute of Psychology of Chinese Academy of Sciences approved the current study. All participants provided written informed consent prior to their participation in the experiments.

### Image Preprocessing

Data pre-processing was performed using the Statistical Parametric Mapping program^1^ (SPM8) and the Data Processing Assistant for Resting-State fMRI^2^ (DPARSF). This included the following: removal of the first 5 volumes, corrections for the intra-volume acquisition time differences between the slices using the Sinc interpolation, corrections for the inter-volume geometrical displacement due to head motion using a 6-parameter (rigid body) spatial transformation, a normalization to the standard Montreal Neurological Institute (MNI) space (resampling voxel size, 3 mm × 3 mm × 3 mm) using the DARTEL approach (Ashburner, 2007), spatial smoothing with a 4-mm full width at a half maximum Gaussian kernel to decrease the spatial noise, and de-trending and temporal band-pass filtering (0.01–0.08 Hz) to reduce the effects of low-frequency drifts and high-frequency physiological noise (Lowe et al., 1998). To remove the head motions for each participant, we performed a nuisance regression of the head motion, using a Friston 24-parameter model (6 head motion parameters, 6 head motion parameters one time point before, and the 12 corresponding squared items) (Friston et al., 1996) with scrubbing (Satterthwaite et al., 2013; Yan et al., 2013a,b; Power et al., 2014). We calculated the mean framewise displacement (FD), which was derived using the Jenkinson’s relative root mean square (RMS) algorithm (Jenkinson et al., 2002). This was used as a covariate in the group analyses of the connectivity–cognition correlations to further control for any residual effects of head movement (Yan et al., 2013a,b; Power et al., 2014). In addition, we performed a nuisance regression of the global signal (the average voxel signal within the SPM *apriori* mask (brainmask.nii) thresholded at 50%, and the white matter and cerebrospinal fluid signals, which were calculated by averaging the voxel signals within the SPM *apriori* masks (white.nii and csf.nii, respectively) thresholded at 99%. The residual volumes were retained for use in the following functional connectivity analysis.

### Measuring the Inter-Individual Variability of Functional Connectivity

To create the regions for the functional connectivity analyses, we parcellated the brain into 116 ROIs, including 90 cerebral regions and 26 cerebellar regions, based on the AAL atlas (Tzourio-Mazoyer et al., 2002). To ensure that only the gray matter voxels within the AAL ROIs were included in the analyses, these ROIs were multiplied by the SPM’s gray matter mask, which was thresholded at 20%, to further remove white matter, cerebrospinal fluid, and other non-brain tissue voxels. The mean time series of each ROI was calculated. Pearson’s linear correlation coefficients (*r* values) were computed between each ROI pair of the averaged time series and subsequently transformed to Fisher *z* values, which yielded a 116 × 116 correlation matrix for each participant. For a given AAL ROI *R_i_* (*i* = 1, 2, … 116), the functional connectivity of the participant, *S_m_* (*m* = 1, 2, … 88), was denoted as a 1 × 115 correlation coefficient vector, *FC*(*S_m_*)*_i_*, in which each element corresponded to its correlation with each of the remaining 115 regions. To quantify the inter-individual variability at *R_i_*, the inter-individual similarity, *FCS_i_* was first calculated as the mean (*E*) of the correlation values between any two functional connectivity vectors of the 88 older participants:

$$FCS_{i}=E[corr(FC{\left(S_{m}\right)}_{i},FC{\left(S_{n}\right)}_{i}],$$

where *m, n* = 1, 2, … 88, and *m*≠*n*.

The inverted similarity (1–*FCS_i_*) was thus defined as the inter-individual variability (*FCV_i_*) of the functional connectivity at *R_i_* (Mueller et al., 2013). This calculation was repeated for all *R_i_* ROIs to derive the spatial distribution of the inter-individual variability of the functional connectivity across the entire brain.

Further, we investigated the inter-individual variability for distinct functional systems in the older participants. Previous functional connectome analyses of the brain architecture indicated the existence of a hierarchical modularity, which is typically represented as intrinsic functional networks (Ferrarini et al., 2009; He et al., 2009; Park and Friston, 2013; Turk-Browne, 2013). Here, we associated the 90 cerebrum regions with five networks, including the sensorimotor and auditory network, visual network, fronto-parietal network related to attention and executive function, default-mode network, and the subcortical network (He et al., 2009), and another 26 regions to the cerebellar network. The inter-individual variability values were averaged across the regions from the same functional network. A one-way analysis of variance (ANOVA) with network as a factor (six networks) followed by *post hoc* pair-wise comparisons were performed to investigate the differences in the inter-individual variability between the different functional networks (Bonferroni corrected for 15 comparisons, threshold at 0.05/15 ≈ 0.0033).

### Linking Inter-Individual Functional Variability to Cognitive Ability

First, we calculated the correlations between functional connectivity and cognitive ability. Individual cognitive performance was assessed using four functional domains, including working memory (indexed by the average *z*-score of DFS and DBS), episodic memory (the *z*-score of PALT), executive function (inverted *z*-score of TMT B-A), and vocabulary (the *z*-score of VFT). In addition, the composite average *z*-score on all tests was considered a measure of individual global cognitive function. Correlation analyses between each functional domain and the global measure and connectivity of all ROI pairs were performed in a subset of participants (*n* = 76). With an emphasis on the overall trend of the connectivity–cognition relationship, we used a liberal threshold of *p* < 0.01 to map the correlation patterns between the cognitive measures and interregional connectivity of all ROI pairs. Age, sex, education level, and the mean head motion FD were considered covariates during the connectivity–cognition correlation analyses. In addition, to further describe the relationship between individual cognition levels to the connectome measures, the number of long-range (Euclidean distance >75 mm between the centroids of the connected regions in stereotactic space), short-range (Euclidean distance ≤ 75 mm) (Achard et al., 2006; Liang et al., 2013), intra-network (connections within the 6 networks mentioned above), and inter-network (connections between the six networks) connections that were significantly related to each cognitive measure were calculated.

Then, to quantify the significance of the functional connectivity of each region with individual cognitive ability in elderly individuals, a cognitive relevance index was defined. It was measured as the number of connections (including the total connections, long-/short-range connections, and inter-/intra-network connections, respectively) that was significantly correlated with all cognitive variables at each ROI.

Finally, to evaluate the cognitive significance of inter-individual variability in connectivity, we examined the correlation between the values of inter-individual variability and the values of cognitive relevance across all the AAL ROIs (*p* < 0.05). We were interested in examining whether a larger inter-individual variability in the brain connectivity would be more cognitively relevant.

### Evaluating Potential Confounding Factors

First, global signal regression (GSR) is a controversial step that may significantly affect the results and conclusions. Recent studies have suggested that GSR can decrease dependence on motion, remove artifactual variance, and provide increased tissue sensitivity (Fox et al., 2009; Satterthwaite et al., 2013; Yan et al., 2013a; Power et al., 2014). However, other studies have demonstrated that GSR may introduce undesirable negative correlations (otherwise largely absent from the connectivity data) that alter inter-individual differences (Fox et al., 2009; Gotts et al., 2013; Saad et al., 2013). In view of these conflicting reports, we included the results without GSR (nGSR) as Supplementary Material for the present study.

Second, different AAL regions vary in regional noise and volume, both of which may potentially drive the inter-individual variability distribution. To rule out these possibilities, we calculated the temporal signal-to-noise ratio (SNR), which was measured as the average signal across time divided by standard deviation across time for each voxel, and averaged the SNR of voxels within each ROI. We also calculated the number of voxels for each ROI to index the volume of each AAL region. A correlation analysis (*p* < 0.05) between the SNR/volume and the inter-individual variability values of the ROIs was examined.

Third, although its size in relation to the entire brain is small, recent studies mapping the cerebellar topographical organization suggest that the cerebellum is functionally heterogeneous (Buckner, 2013). Therefore, cerebellar ROIs may be more prone to contain functionally diverse gray matter compared to that of other ROIs. To rule out this potential confound, a connectivity atlas of the cerebellum, which was adopted by a previous study (Buckner et al., 2011) with large data set (*N* = 1000) to calculate the functional connectivity of different cerebellar regions with neocortical network, was used to perform an additional analysis. We chose the 17-network parcellation atlas of the cerebellum. The voxels assigned to the same network were considered as one ROI; thus, the 17 ROIs from Buckner et al. (2011) were used to replace the 26 AAL cerebellar ROIs, and to recalculate the inter-individual functional variability in the brain. This allowed us to rule out the possibility that high functional heterogeneity in the cerebellum may influence the variability estimation.

Finally, to further confirm the robustness of the result with regard to functional inter-individual variability in the elderly, we validated the result by analyzing an independent replication resting-state fMRI dataset (*N* = 49; 12 men and 37 women; 67.1 ± 4.8 years; range: 60–76 years of age). The data were acquired using a Philips Achieva 3.0-T MRI scanner (Philips Healthcare, Andover, MA) at the MRI Center of the First Hospital of Hebei Medical University of China. Functional images were collected using an EPI sequence with TR = 2000 ms, TE = 30 ms, flip angle = 90°, FOV = 200 mm × 200 mm, thickness = 3.6 mm, matrix = 112 × 112; in-plane resolution = 1.786 × 1.786, 33 axial slices, and 200 volumes. T1-weighted MPRAGE image was collected with the following parameters: 176 slices; matrix = 256 × 256; voxel size = 1 mm × 1 mm × 1 mm; TR = 1900 ms; TE = 2.2 ms; flip angle = 9°. The individual variability of functional connectivity in this dataset was estimated using the same procedure as described above.

## Results

### Inter-Individual Variability in Functional Brain Connectivity

**FIGURE 1:**
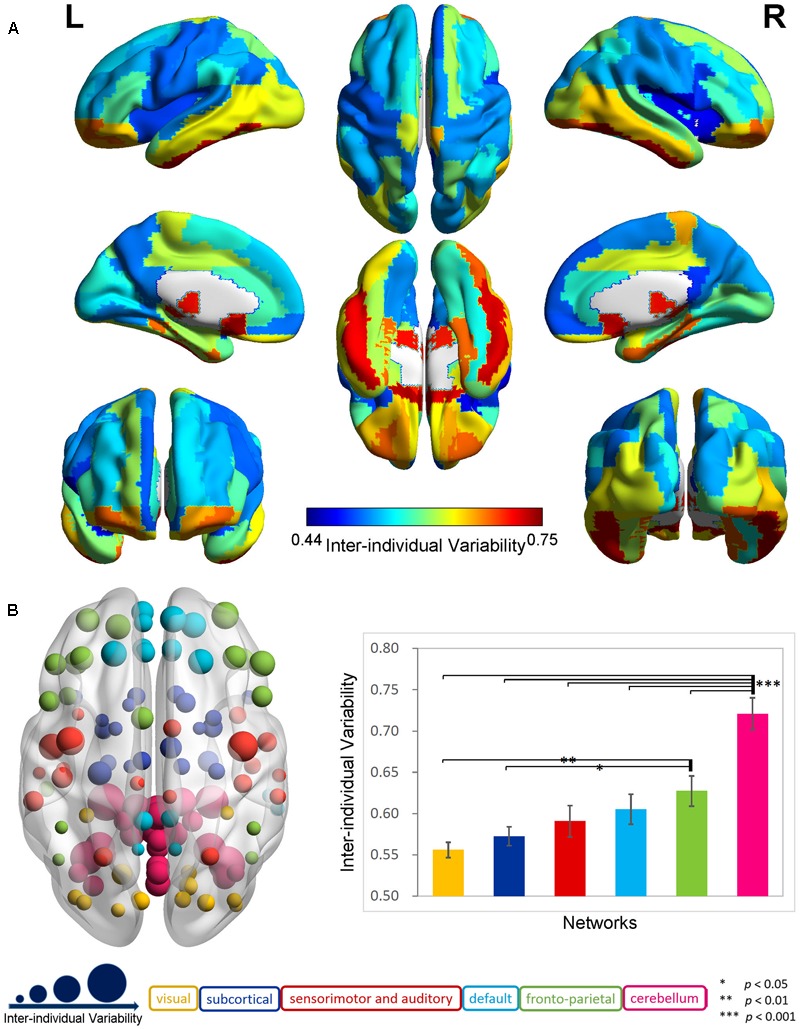
Inter-individual difference in functional brain connectivity in elderly individuals. **(A)** Distribution of inter-individual functional variability in the cerebrum. The inter-individual variability values for the 90-automated anatomical labeling (AAL) cerebral regions were mapped onto the cortical surfaces using varied colors. **(B)** Inter-individual variability in functional networks. The left axial map shows the inter-individual variability in the functional connectivity for 116 AAL regions, which are rendered as color-coded nodes, according to the functional networks (He et al., 2009). The nodes are located at the center of these regions, and the nodal size is proportional to the level of the inter-individual variability. The right histogram plots the averaged inter-individual variability values and the standard errors for the functional networks, which are displayed as color-coded bars in the corresponding color applied to the nodes.

The exploration of the whole-brain functional connectivity in 116 AAL regions indicated a highly uneven distribution pattern for inter-individual variability in the 88 older participants (Supplementary Figure 1). There was an overall tendency that the inter-individual functional variability increased from the primary areas to the subcortical structures and association cortex to the cerebellum across the whole brain. The mean variability in the cerebellum (0.72 ± 0.10) was significantly larger (two-sample *t*-test, *p* < 0.0001) than that in the cerebral regions (0.59 ± 0.07). In the cerebrum (**Figure 1A**), the inter-individual difference in functional connectivity was higher in the frontal and parietal cortices; pre- and post-central gyri; anterior, middle and posterior cingulated gyri; parahippocampus; hippocampus; and amygdala and lower in the occipital, temporal, and other subcortical regions.

The analyses in the six specific functional systems (**Figure 1B**) further highlighted a gradual increase in the functional variability from the visual, subcortical, and sensorimotor and auditory networks to the default and fronto-parietal networks, and to the cerebellar network. The ANOVA revealed a significant main effect of network in the functional variability (*p* < 0.001). The *post hoc* comparisons demonstrated that the mean inter-individual variability in the cerebellar network was significantly larger (*p* < 0.001) than that of each of the other five networks at a Bonferroni-corrected threshold of *p* = 0.0033 (0.05/15). The fronto-parietal network exhibited a trend toward a higher variability compared with the visual network (*p* < 0.01) and subcortical network (*p* < 0.05).

### Connectivity–Cognition Correlation

**FIGURE 2:**
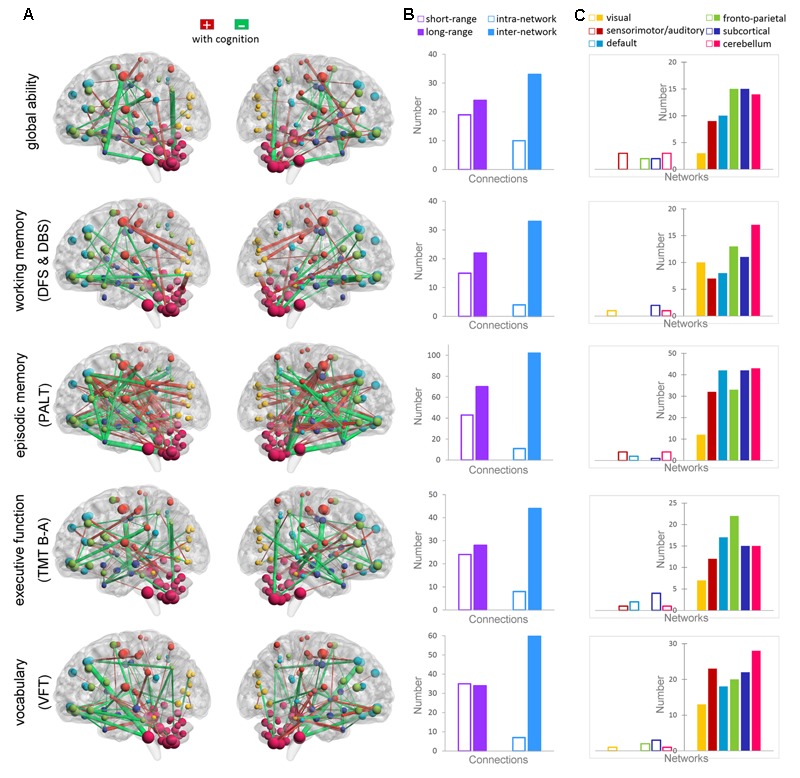
Correlations between functional connectivity and the cognitive measures of global ability, working memory (DFS and DBS), episodic memory (PALT), executive function (TMT B-A), and vocabulary ability (VFT). **(A)** Maps showing significant correlations between the connectivity and cognitive ability (*p* < 0.01). The connections that positively correlated with cognition are shown in red, whereas the connections that negatively correlated with cognition are shown in green. The thickness of the connections is proportional to the connectivity–cognition correlation coefficients. **(B)** The bars show the total number of short-range and long-range connections, as well as the intra-network and inter-network connections that are correlated with each cognitive domain. **(C)** The bars show the total number of connections within each functional network (transparent bars) and the total number of connections with other networks (non-transparent bars) that are correlated with each cognitive domain.

**Figure 2** shows the Pearson correlations of the connectivity of all ROI pairs with individual global cognitive function and the four specific cognitive domains (*p* < 0.01, uncorrected). The largest number of connections from the superior and orbital prefrontal cortex and the cerebellum consistently correlated with individual scores in global cognition and in the four specific cognitive domains. Further, the functional connectivity of the following connections were related to the four cognitive measures: (1) from the middle, anterior, and posterior cingulate; hippocampus; parahippocampus; amygdala; and precentral gyrus for working memory (DFS and DBS); (2) from the middle temporal pole, middle temporal gyrus, postcentral gyrus, precuneus, thalamus, parahippocampus, hippocampus, anterior and posterior cingulate, and putamen for episodic memory (PALT); (3) from the middle temporal gyrus, middle temporal pole, anterior cingulate, inferior parietal lobule, and parahippocampus for executive function (TMT B-A); and (4) from the fusiform, supramarginal gyrus, angular gyrus, middle temporal gyrus, middle temporal pole, and hippocampus for vocabulary (VFT).

**FIGURE 3:**
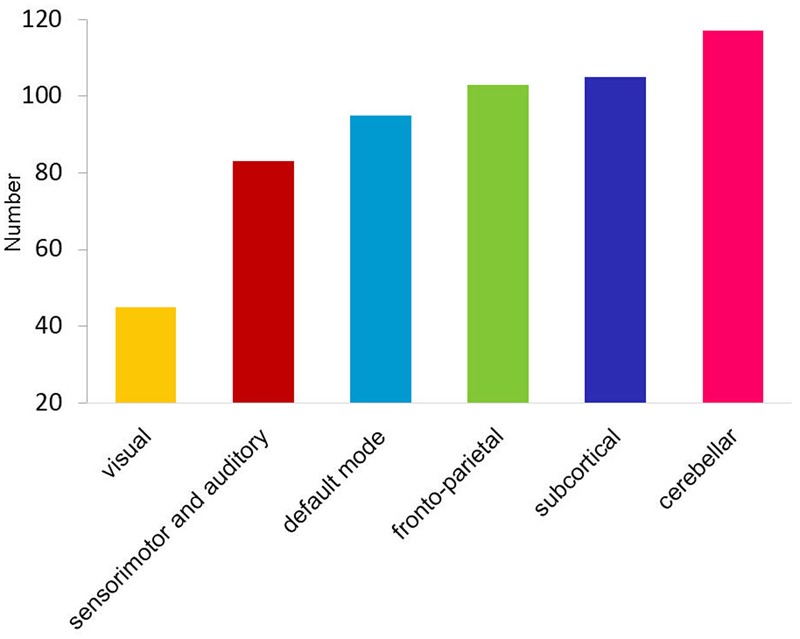
Bar graph shows the ascending order of the total number of cognition-related inter-network connections for each functional network.

Long-range and inter-network connectivity accounted for a considerable proportion of connections that predicted individual cognition (**Figure 2B**). There were more long-range connections than short-range connections (56.7% vs. 43.3% in total), which correlated with both global measures and specific measures, except for the vocabulary score. Moreover, inter-network connections accounted for 87.3% of all the connections that correlated with the cognitive measures in the whole brain, and consistently preponderated over the intra-network connections when the six functional networks were separately analyzed (**Figure 2C**). We also summarized the total number of inter-network connections that correlated with the four specific cognitive measures for each network. Interestingly, we found that the six networks were in the same variability rank order, except for the subcortical network, which moved up to second place (**Figure 3**).

**FIGURE 4:**
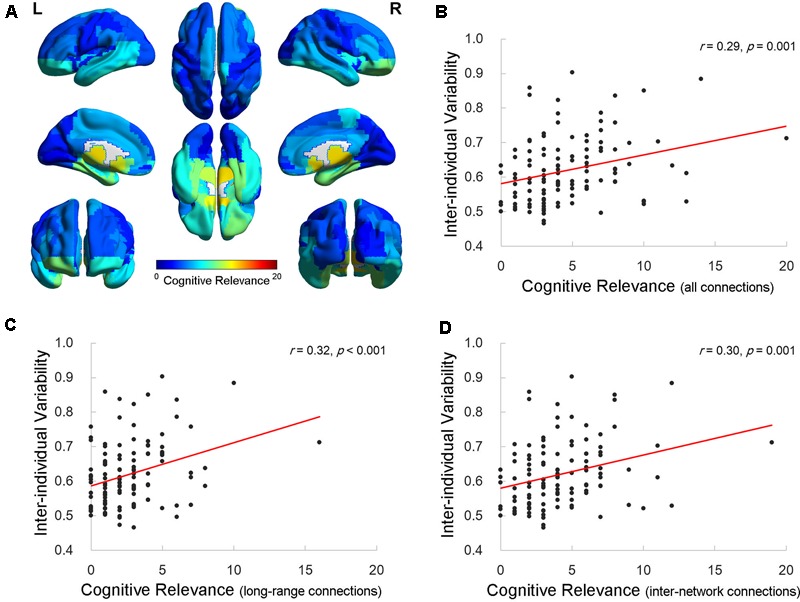
Relationship between inter-individual variability and the cognitive relevance of functional connectivity. **(A)** The cognitive relevance map of AAL cerebral regions. Each AAL regions of interest (ROI) was color coded as the total number of connections that are correlated with four specific cognitive domains. **(B–D)** The scatter plots show the correlation between the inter-individual variability and cognitive relevance as indexed by the total number of cognition-related connections **(B)**, long-range connections **(C)**, and inter-network connections **(D)** across 116 AAL ROIs, respectively. Each dot represents one ROI from AAL.

Finally, we calculated the cognitive relevance, which was indexed by the number of connections that were significantly correlated with the cognitive measures, for each AAL ROI. The distribution map for the cognitive relevance (**Figure 4A**) was similar to the inter-individual functional connectivity variability map (**Figure 1A**). The correlation analysis revealed that the value of the inter-individual functional variability was significantly correlated with the cognitive relevance across the 116 ROIs (Pearson correlation *r* = 0.29, *p* = 0.001; **Figure 4B**). Regions with higher inter-individual functional connectivity variability demonstrated more connections that correlated with cognitive performance. More interestingly, when examining the number of long-/short-range and inter-/intra-network connections, the value of the inter-individual variability significantly correlated with the degree of cognitive relevance for the long-range (Pearson correlation *r* = 0.32, *p* < 0.001; **Figure 4C**) and inter-network (Pearson correlation *r* = 0.30, *p* = 0.001; **Figure 4D**) connectivity across all ROIs. There was no significant correlation between the inter-individual variability and the short-range (Pearson correlation *r* = 0.10, *p* = 0.27) or intra-network (Pearson correlation *r* = 0.16, *p* = 0.09) connectivity cognitive relevance measures in the brains of elderly individuals.

### Impact of Potential Confounds

First, we re-calculated the functional inter-individual variability without removing the global signal in the preprocessing. Variability maps, estimated with (**Figure 1**) and without (Supplementary Figures 1, 2) GSR, demonstrated a highly similar pattern (Pearson correlation *r* = 0.92, *p* < 0.0001). The cerebellum retained the largest mean inter-individual variability compared to that of cerebral regions (two-sample *t*-test, *p* < 0.0001). The network-level variability also consistently demonstrated significant statistical difference for the functional variability among the networks (*p* < 0.001), with gradually increased variability occurring in the subcortical network, then the primary networks (i.e., visual, sensorimotor, and auditory networks), to the association networks (i.e., default and fronto-parietal networks), and to the cerebellar network (Supplementary Figure 2B). However, as expected, the GSR largely affected the connectivity–cognition correlations, such that the GSR preprocessing introduced more negative correlations (**Figure 2**) than the nGSR preprocessing (Supplementary Figure 3). As an overall trend, this was consistent with the GSR results regarding the inter-network connectivity, especially for the connections from the superior and orbital prefrontal cortex, hippocampus, and the cerebellum predominating individual cognitive ability. It is important to note that the retention of the global signal diminished the correlation between the long-range connections and cognition, with a larger proportion of the long-range connections only found in the global measure and vocabulary score. In addition, in the nGSR condition, the relationship between the value of the inter-individual functional connectivity variability and the cognitive relevance across all ROIs disappeared (Pearson correlation *r* = –0.13, *p* = 0.17; Supplementary Figure 4).

Next, we calculated the correlation between the regional SNR/size and the inter-individual functional connectivity variability values across all ROIs, to exclude the possibility that the ranking of the regional inter-individual variability was primarily driven by potential noise and size effects. The rank of the inter-individual variability derived with GSR was not influenced by the regional noise or size (*p* > 0.05). The supplementary nGSR result of the inter-individual variability, however, correlated significantly with the regional SNR (Pearson correlation *r* = 0.34, *p* < 0.01).

Third, to rule out the possibility that high functional heterogeneity in the cerebellar ROIs influenced the variability estimation, we used the 17-network parcellation atlas of the cerebellum (Buckner et al., 2011) to replace the 26 cerebellar AAL ROIs, which allowed us to perform an additional analysis of the inter-individual functional connectivity variability. Consistent with our findings using the cerebellar AAL ROIs, the additional analysis demonstrated that 5 of the 17 cerebellar ROIs ranked highly for the inter-individual functional variability in the brain. The mean variability of the 17 cerebellar ROIs (0.66 ± 0.12) was significantly larger (two-sample *t*-test, *p* = 0.0001) than that of the cerebral ROIs (0.58 ± 0.07). No significant differences were found for the inter-individual functional connectivity variability in the cerebellum between the two different atlases (two-sample *t*-test, *p* = 0.09).

Finally, the robust validation analysis in an independent dataset further confirmed the distribution of inter-individual functional connectivity variability in the brains of elderly people. The distribution patterns in both datasets were highly similar (Pearson correlation *r* = 0.61, *p* < 0.0001). Further, the cerebellum had maximal inter-individual variability (0.72 ± 0.11), and the cerebrum demonstrated gradually increased inter-individual variability from the visual (0.61 ± 0.06), subcortical (0.62 ± 0.05), and sensorimotor and auditory (0.63 ± 0.09) networks to the fronto-parietal (0.64 ± 0.09) and default (0.69 ± 0.09) networks.

## Discussion

There is fairly extensive research regarding the relationship between changes in brain connectivity and a broad range of cognitive decline and neuropsychiatric symptoms in aging populations (Hedden and Gabrieli, 2004; Reuter-Lorenz and Lustig, 2005; Andrews-Hanna et al., 2007; Wang et al., 2007, 2015; Bishop et al., 2010; Grady, 2012; Tomasi and Volkow, 2012; Ferreira and Busatto, 2013; Li et al., 2013, 2015; Fornito et al., 2015). Although these studies strongly supported the notion that brain connectivity is an important determinant of cognitive aging, the contribution of person-to-person variation remained unclear. Thus, the present study aimed to bridge the gap in knowledge of how individual variability of functional connectivity and the inter-individual differences affect the cognitive ability of elderly individuals. Our novel study systematically mapped the distribution of individual functional variability on a whole-brain scale, which facilitated understanding of how inter-individual variability differs between different brain areas in the older adults. Further, we demonstrated that the inter-individual variability mapping has important cognitive significance. These findings may thus contribute a valuable reference or evidence for future cognitive aging studies.

### Inter-Individual Functional Variability in Elderly Individuals

Functional connectivity in the cerebral cortex indicated that there was higher inter-individual variability in the frontal and parietal cortices, and the pre- and post-central gyri, while there was lower variation in the occipital and temporal regions in elderly individuals. The cortical variability generally aligned with the results from Mueller et al. (2013) who conducted a study of the inter-individual differences in cortical connectivity in 25 healthy adults. In the current study, we expanded this previous work to include a global analysis of the brain in a large sample of older adults. Our findings indicate that inter-individual variability was the largest in the cerebellum, followed by the association regions that largely constitute the fronto-parietal and default mode networks, as well as some subcortical regions, especially the hippocampal formation. Primary regions, including visual and sensorimotor networks, and other subcortical structures exhibited minimal variability among individuals.

It is not surprising that the functional connectivity in the prefrontal and parietal cortices and the relevant fronto-parietal and default mode networks demonstrated major individual variations in the cortex, because extensive evidence suggests these association regions and network connections are the selective targets of aging effects (Andrews-Hanna et al., 2007; Grady, 2012; Tomasi and Volkow, 2012; Ferreira and Busatto, 2013). The cerebellum has not been substantially investigated in most aging studies. However, there is increasingly converging evidence to suggest that the cerebellum is connected to cerebral association regions, including the prefrontal and posterior parietal cortices, and subcortical structures, including the vestibular nuclei and basal ganglia. Therefore, the cerebellum can contribute to a wide variety of functional domains and neuropsychiatric diseases (Stoodley and Schmahmann, 2009; Bostan et al., 2013; Buckner, 2013). Wagner et al. (2017) recently observed that the cerebellar granule cells could encode reward expectation, suggesting that the cerebellum was involved in cognitive processing (Wagner et al., 2017). Further, in a recent review of multidisciplinary findings, Sokolov et al. (2017) suggested a cerebro-cerebellar loop to explain the involvement of the cerebellum in higher cognitive functions, including attention, language, memory, and social cognition (Sokolov et al., 2017). We identified that the largest inter-individual variability resides in the cerebellum, further indicating that it is a noteworthy region for future aging studies. Additional potential studies include the exploration of how the cerebellum is mediated by the prefrontal and parietal regions in the association functional networks, which would provide a better understanding of its role in aging.

Several potential causes may underlie the distribution of the inter-individual variability in the brain functional connectivity of older individuals. First, the hemodynamic MRI signal is triggered by the metabolic demands of neuronal activities (Heeger and Ress, 2002). The variability map of functional connectivity is consistent with the previous metabolic topography of normal aging, as investigated by positron emission tomography; this technique demonstrated covariant metabolic changes in the prefrontal cortex, lateral temporal and parietal cortices, cerebellum, and basal ganglia (Moeller et al., 1996; Chiaravalloti et al., 2014). Thus, we speculated that the inter-individual variability in the functional connectivity had a physiologically reasonable metabolic basis. Second, our findings may be, in part, a functional consequence of the individual heterogeneity in brain structure morphology that occurs with aging. MRI volumetric studies have demonstrated heterogenic aging patterns across structures regarding neuroanatomical volume loss (Jernigan et al., 2001; Walhovd et al., 2005). Jernigan et al. (2001) observed that the cerebellum exhibited the same striking degree of gray matter reduction with aging as the frontal lobes, and exhibited a more accelerated volume loss than the hippocampus (Jernigan et al., 2001). In addition, other anatomical profiles, such as its cortical folding, thickness, and white matter fiber tracts, may also contribute to the individual differences in the functional correlations (Kanai and Rees, 2011; Mueller et al., 2013; Karama et al., 2014). For example, diffusion tensor imaging of white fiber tracts demonstrated that the variability of aging effects was also regionally complex; this was indicated by a gradient increase in the white matter deficits from the posterior to anterior cortex segments, but also by a greater impairment in the cerebellum (Davis et al., 2009; Bennett et al., 2010). Third, the diverse dynamics and heterogeneous distributions of neurons, as well as the selective vulnerability of synapses and neurons during aging, may also promote individual differences in functional connectivity (Morrison and Hof, 1997; Zhao et al., 2008; Bishop et al., 2010; Urban and Tripathy, 2012; Mejias and Longtin, 2014). Although the cerebellum only accounts for approximately 10% of the total brain weight, it accounts for half of its neurons. Thus, the cerebellum would naturally exhibit more variations due to its densely packed neuronal assembly. Finally, genetic and plasticity factors play critical roles in the inter-individual variability in brain connectivity (Mueller et al., 2013; Toro et al., 2014). Genes determine the individual differences in the evolutionarily recent association cortex, specifically in the prefrontal region (Thompson et al., 2001), where the gene expression patterns exhibit substantially greater heterogeneity in middle-old aged populations (Lu et al., 2004; Bishop et al., 2010). Furthermore, the prefrontal cortex and cerebellum are the final structures to achieve maturity, but are also the first structures to undergo involution in later life (Wang and Zoghbi, 2001; Hogan et al., 2011). This protracted development and prolonged degeneration processes can continue to accumulate deeper and more complex inter-individual variations via environment- and lifestyle-dependent neural plasticity.

### Cognitive Relevance of the Inter-Individual Connectivity Variability

The connectivity–cognition correlation suggested that the connections that are related to cognitive ability lay mainly in regions with large inter-individual connectivity differences, including the prefrontal cortex, hippocampal formation, inferior parietal gyrus, middle temporal pole, middle temporal gyrus, and cerebellar regions. The prefrontal, parietal, and temporal regions are the uppermost components of the fronto-parietal and default mode networks, which support high-level cognition. Numerous molecular and neuroimaging studies have repeatedly confirmed their role in cognitive aging, such as in memory, attention, and executive function decline (Hedden and Gabrieli, 2004; Burke and Barnes, 2006; Andrews-Hanna et al., 2007; Bishop et al., 2010; Grady, 2012; Nyberg et al., 2012; Tomasi and Volkow, 2012; Ferreira and Busatto, 2013; Karama et al., 2014). The hippocampal formation is also particularly vulnerable to the aging process. Here, we demonstrated that the functional connectivity of the hippocampus and parahippocampus is correlated with all four cognitive measures, including working memory (DFS and DBS), episodic memory (PALT), executive function (TMT B-A), and vocabulary (VFT). Our results are consistent with a recent meta-analysis of 114 fMRI studies of older adults, which suggested a set of regions that are remarkably involved in cognitive aging; these included the frontal gyrus, parahippocampal gyrus, fusiform gyrus, precentral gyrus, and functional networks, especially the fronto-parietal and default networks (Li et al., 2015).

Long-range and inter-network connections appeared to dominate cognitive ability differences among older adults. Long-range connections are well-known for their key role in efficient brain-wide information processing and functional integration of diverse cognitive functions (Jbabdi et al., 2013; Park and Friston, 2013). Previous evidence demonstrated that the long-range connections in the default and fronto-parietal attention networks are selectively vulnerable to aging and are susceptible to early Alzheimer’s disease, compared to that of the short-range connections (Andrews-Hanna et al., 2007; Tomasi and Volkow, 2012; Li et al., 2013; Wang et al., 2013; Sala-Llonch et al., 2014). Recently, Fjell et al. (2015) found extensive changes in inter-network functional connectivity across multiple cortical networks that were related to a decline in episodic memory with aging (Fjell et al., 2015). It is also interesting to note that although the subcortical network ranked lower for the average inter-individual variability, some specific regions with larger inter-individual differences, including the hippocampal formation, thalamus, caudate, and amygdala, have considerable connections to regions in other networks that are involved in cognition. Therefore, this may suggest that this network, specifically some specific regions, needs to be considered as having a role in cognitive function through its interactions with other cortical and cerebellar networks.

Importantly, we found larger inter-individual variation of functional connectivity was significantly correlated with higher cognitive relevance, in terms of the number of cognition-correlated connections. This relationship suggested that the functional connectome was a major root of individual behavior differences. Moreover, we demonstrated that the correlation between the inter-individual variability and the cognitive relevance of functional connectivity was specific to long-range and inter-network connections. Given the role of long-range and inter-network connections in cognitive performance, this finding further indicated that regions and networks with large inter-individual variability deserve attention in future studies. Thus, these results provide a new perspective for understanding cognitive aging. Currently, most studies are conducted by first assigning participants to different groups, and then exploring differences in the averaged brain activity signals among the groups. In these studies, inter-individual differences in brain function are essentially neglected, or simplified to group differences (Mohr and Nagel, 2010), limiting the full understanding of cognitive aging. Here, the mapping of the inter-individual functional connectivity variability and its correlation with cognition suggested regions and connections, which are typically overlooked but important to cognitive aging studies. For example, the cerebellum showed the largest inter-individual variability and was correlated with diverse cognitive domains. In fact, several studies investigating the role of the cerebellum in aging has emerged. Increasing evidence has indicated that the cerebellum is involved in frontally based functional decline in elderly individuals (Sullivan and Pfefferbaum, 2006; Hogan et al., 2011; Bernard and Seidler, 2014). Future studies should investigate how the prefrontal cortex interacts with the cerebellum, subcortical areas, and other cortical regions to contribute to the inter-individual differences seen with aging. This would be particularly important to distinguish the connectivity–cognition associations that are specific to aging from the inherent general relationships across the lifespan. For example, previously the prefrontal cortex has been overwhelmingly emphasized in cognitive aging. However, a previous molecular genetic expression study (Erraji-Benchekroun et al., 2005) and a recent cortical thickness study (Karama et al., 2014) have stressed that the prefrontal cortex is in fact linked closely with diverse cognitive abilities throughout the human life-span. The mapping of inter-individual variability thus brings a new perspective to future studies that seek key areas affected by cognitive aging. It will also be exciting to investigate inter-individual connectivity variability and its cognitive importance over time to further understand inter-individual differences in the trajectories of cognitive aging and specific diseases, such as AD.

### Limitations

A few limitations of the present study must be noted. First, given the complex and controversial involvement of the GSR in fcMRI studies, we included results both with and without GSR. The GSR was expected to influence the results. The inter-individual variability rank estimated from data with GSR appeared to be more sensitive to the noise than the nGSR results, despite a similar inter-individual ROI variability ranking with both strategies. In addition, the GSR produced negative biased correlations between the individual connectivity and cognitive measures and magnified the proportion of long-range connections that were correlated with performance, compared to that of the nGSR results. However, both suggested an important role for the inter-network connections in cognitive aging. The influence of the regression of global signal in the present result needs to be carefully considered. Second, we noted that as the present study was confined to the estimation of the distribution of inter-individual functional variability of older adults, intra-individual variations were not considered. Variations in the intra-individual functional connectivity may be caused by measurement instability due to technical noise or changes of mental and biological states (Mueller et al., 2013). A recent study conducted by Chen et al. (2015) depicted the pattern of intra-individual functional variability in the brain of young adults with the use of ten repeated fMRI measurements. Another factor is the temporal moment-to-moment variation within an individual’s BOLD signal, which has also been suggested to have predictive significance in relation to cognitive function and various clinical conditions (Garrett et al., 2013). It is necessary for future studies to investigate the distribution characteristic of these intra-individual variations and examine their cognitive correlations with aging and to further investigate how these variations may interact with inter-individual variability. Third, we acknowledge that the connectivity–cognition correlation was not corrected for multiple comparisons. This is because the focus of this study was not to report which regional connections were significantly correlated with cognitive performance. We used a threshold of *p* < 0.01 (corresponding to *r* > 0.30) to define the cognitive relevance index for each region, which helped disclose an overall relationship between functional connectivity variability and cognitive association across all brain areas. The definition of “cognitive relevance index” in our study was similar to that of other fMRI connectivity measurements, such as “functional connectivity density” or “degree centrality,” which is usually calculated as the number of correlated connections at a liberal correlation coefficient threshold (e.g., *r* = 0.25), without multiple corrections on the correlations between mass voxels (Buckner et al., 2009; Weng et al., 2016). Fourth, the AAL atlas we used to calculate functional connectivity was defined on the basis of anatomical features. Although the use of an alternative connectivity atlas of cerebellum did not change the cerebellar rank in inter-individual variability, the influence of the ROI definition from using the AAL cannot be fully excluded. As the division of brain regions, as well as their functional characteristics, remains controversial, future studies of data-driven parcellation of brain regions and networks would present more precise estimation of inter-individual variability in elderly individuals. Finally, the current study was focused on mapping a general profile of inter-individual variability in an older population. No attempt was made to examine factors, such as the age of participants, that influence inter-individual variability. Thus, further studies are required to investigate the effect of age, as well as other environmental or genetic factors, that can influence individual functional variability.

## Conclusion

In the current study, we delineated a map of inter-individual variability in whole-brain functional connectivity for older adults. These results revealed gradually increased variability from the primary regions (including the visual, sensorimotor, and auditory networks), to specific subcortical structures, particularly the hippocampal formation, and the prefrontal and parietal cortices that largely constitute the default mode and fronto-parietal networks, and the cerebellum. The connectivity–cognition results further stressed a crucial function for long-range and inter-network connections in inter-individual cognitive performance. Moreover, the associations between inter-individual variability and the cognition relevance of functional connectivity provide a new perspective for investigating the mechanisms underlying cognitive aging and relevant diseases.

## Author Contributions

Conceived and designed the experiments: RL, JL. Performed the experiments: SY, XZ, WR, JY, PW, ZZ, Y-NN, XH. Analyzed the data and wrote the paper: RL.

## Conflict of Interest Statement

The authors declare that the research was conducted in the absence of any commercial or financial relationships that could be construed as a potential conflict of interest.

## Footnotes

1 http://www.fil.ion.ucl.ac.uk/spm

2 http://www.rfmri.org
