# Supplementary material for: Linking Inter-Individual Variability in Functional Brain Connectivity to Cognitive Ability in Elderly Individuals
Source: Front Aging Neurosci. 2017 Nov 21;9:385. doi: 10.3389/fnagi.2017.00385 (PMC5702299; doi:10.3389/fnagi.2017.00385)
Supplement: Supplementary file 1 [file Data_Sheet_1.DOCX]

**Supplementary Figure Legends**

**Supplementary Fig. 1.** Bar graph in descending order for values of inter-individual functional connectivity variability in 116 AAL regions. (A) The variability rank of AAL regions determined from GSR-based data. (B) The variability rank of AAL regions determined from nGSR-based data.

**Supplementary Fig. 2.** Inter-individual difference in functional brain connectivity in elderly individuals based on data without GSR. (A) Distribution of inter-individual functional variability in the cerebrum. The inter-individual variability values for the 90 AAL cerebral regions were mapped onto the cortical surfaces with varied colors. (B) Inter-individual variability in functional networks. The left axial map shows the inter-individual variability in functional connectivity for 116 AAL regions, which are rendered as color-coded nodes, according to the functional networks ([He et al., 2009](#_ENREF_28)). The nodes are located at the center of these regions, and the nodal size is proportional to the level of the inter-individual variability. The right histogram plots the averaged inter-individual variability values and the standard errors for the functional networks, which are displayed as color-coded bars in the corresponding consistent color applied to the nodes.

**Supplementary Fig. 3.** Correlations between functional connectivity and the cognitive measures of global ability, working memory (DFS and DBS), episodic memory (PALT), executive function (TMT B-A), and vocabulary (VFT) ability based on data without GSR. (A) Maps showing significant correlations between connectivity and cognitive ability(*p*< 0.01). Connections that positively correlated with cognition are shown in red, whereas the connections that negatively correlated with cognition are shown in green. The thickness of the connections is proportional to the connectivity-cognition correlation coefficients. (B) The bars show the total number of short-range and long-range connections, as well as the intra-network and inter-network connections that are correlated with each cognitive domain. (C) The bars show the total number of connections within each functional network (transparent bars) and the total number of connections with other networks (nontransparent bars) that are correlated with each cognitive domain.

**Supplementary Fig. 4.** Relationship between inter-individual variability and the cognitive relevance of functional connectivity based on data without GSR. (A) The cognitive relevance map of AAL cerebral regions. Each AAL ROI was color coded as the total number of connections that are correlated with four specific cognitive domains. (B) The scatter plots show the correlation between the inter-individual variability and cognitive relevance as indexed by the total number of cognition-related connections. Each dot represents one ROI from AAL.
