# Supplementary figures and images for: Linking Inter-Individual Variability in Functional Brain Connectivity to Cognitive Ability in Elderly Individuals
Source: Front Aging Neurosci. 2017 Nov 21;9:385. doi: 10.3389/fnagi.2017.00385 (PMC5702299; doi:10.3389/fnagi.2017.00385)

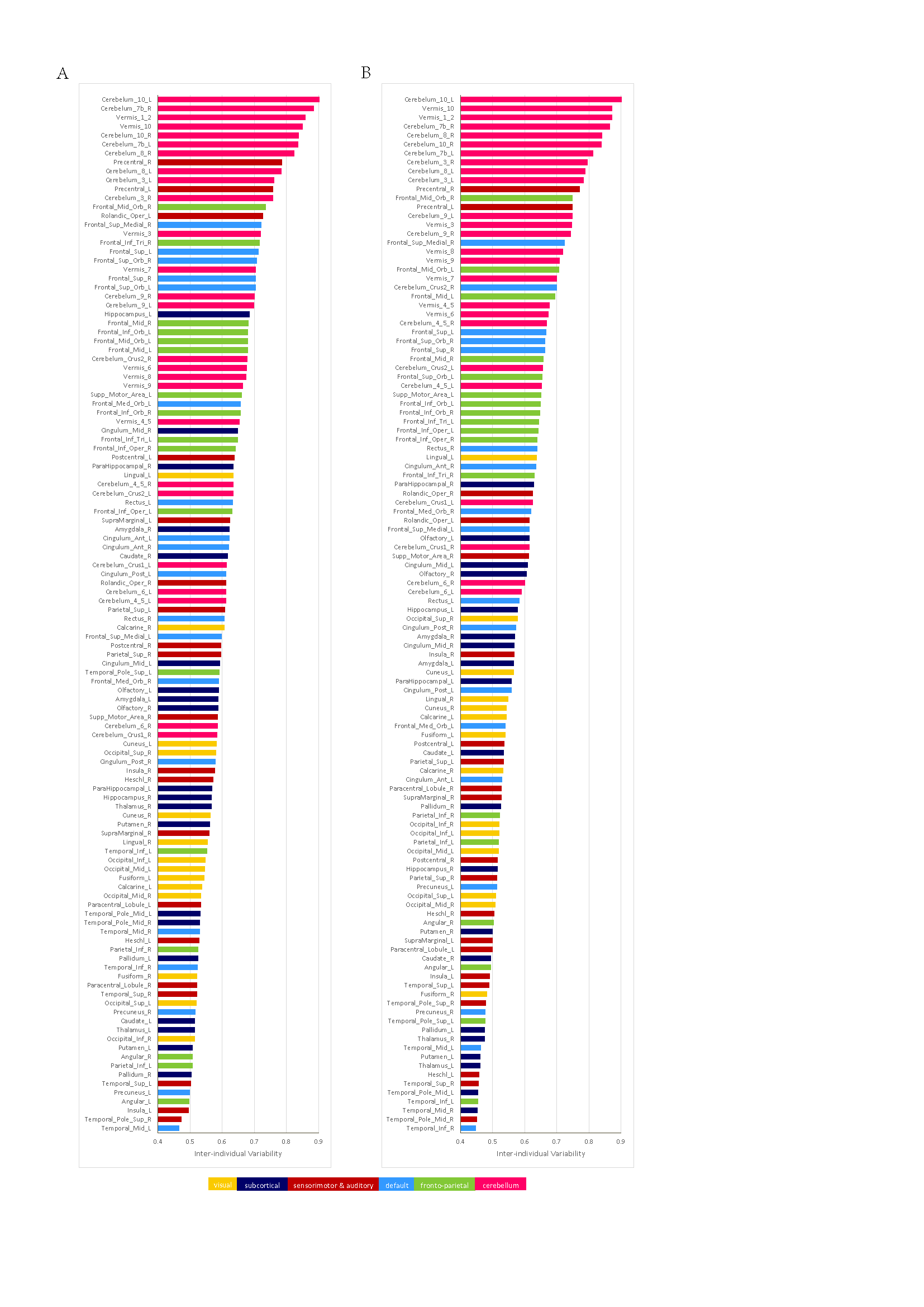

Supplement: Supplementary file 2 [file Image_1.PNG]

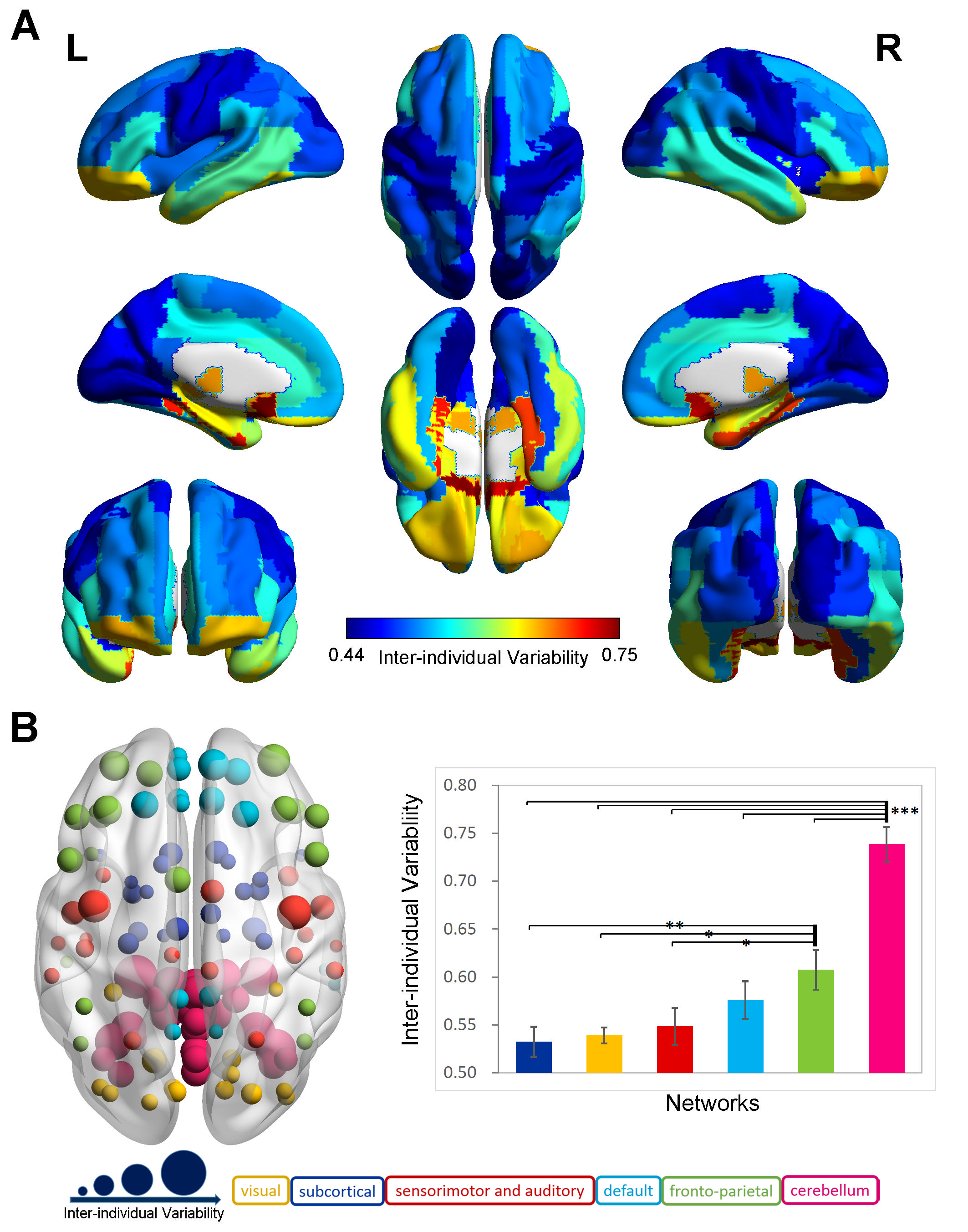

Supplement: Supplementary file 3 [file Image_2.TIF]

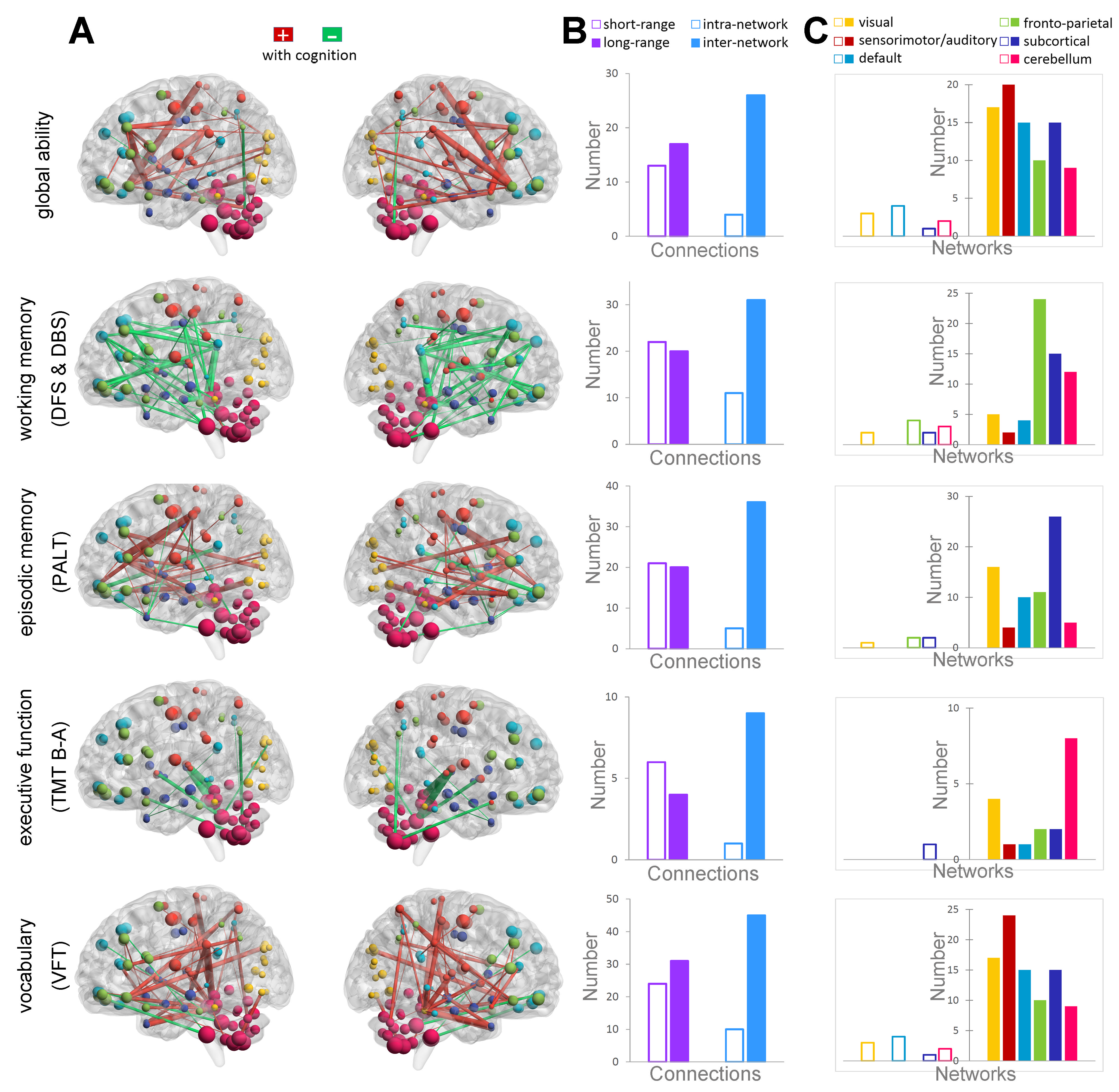

Supplement: Supplementary file 4 [file Image_3.TIF]

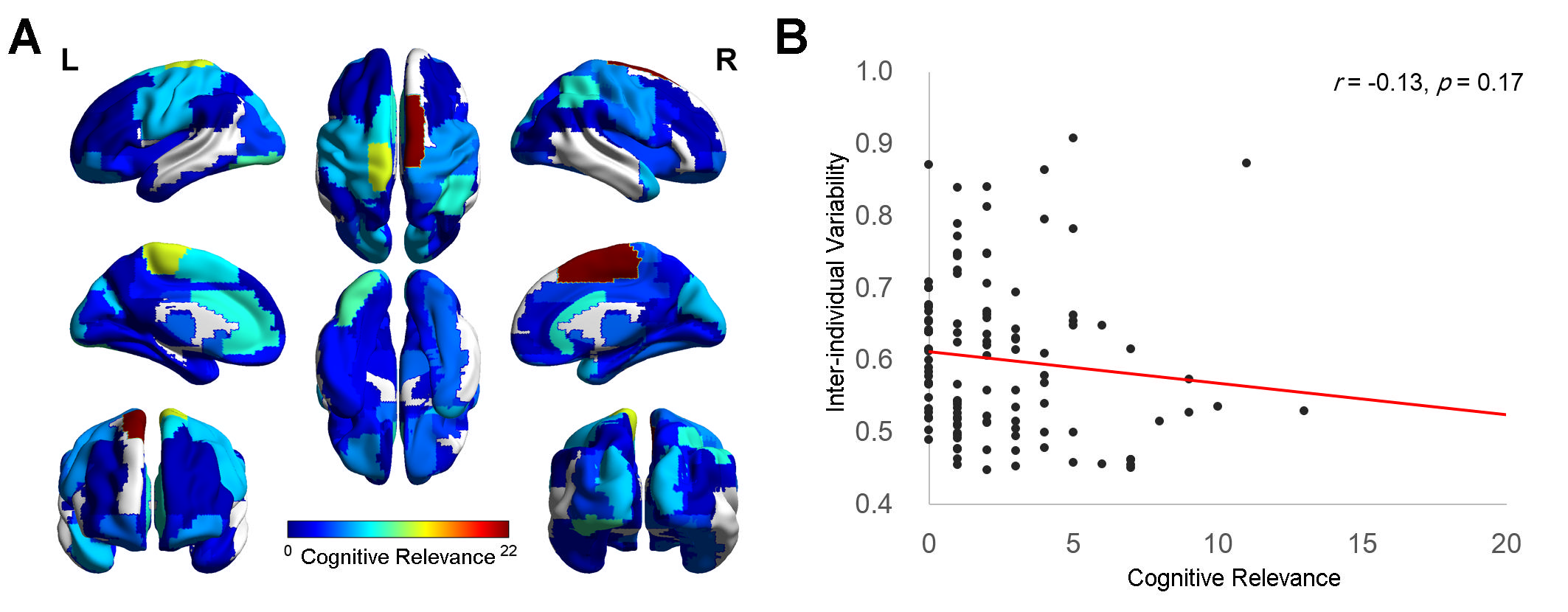

Supplement: Supplementary file 5 [file Image_4.TIF]
